# Aquatic cycling—What do we know? A scoping review on head-out aquatic cycling

**DOI:** 10.1371/journal.pone.0177704

**Published:** 2017-05-16

**Authors:** Stefanie Rewald, Ilse Mesters, Antoine F. Lenssen, Jens Bansi, Johan Lambeck, Rob A. de Bie, Benjamin Waller

**Affiliations:** 1 Department of Epidemiology, Faculty of Health, Medicine and Life Sciences, Care and Public Health Research Institute (CAPHRI), Maastricht University, MD Maastricht, The Netherlands; 2 Department of Physical Therapy, Maastricht University Medical Center, Maastricht, The Netherlands; 3 Department of Sports Therapy, Kliniken-Valens, Rehabilitationsklinik Valens, Valens, Switzerland; 4 Faculty of Kinesiology and Rehabilitation Sciences, KU Leuven, Leuven, Belgium; 5 Faculty of Sport and Health Sciences, University of Jyväskylä, Jyväskylä, Finland; Nanyang Technological University, SINGAPORE

## Abstract

Over the past few years, aquatic cycling has become a trending fitness activity. However, the literature has not been reviewed exhaustively. Therefore, using scoping review methodology, the aim of this review was to explore the current state of the literature concerning aquatic cycling. This study specifically focused on study designs, populations and outcomes. A comprehensive search of seven databases (PubMed, MEDLINE, Cinahl, Embase, PEDro,Web of Science, WorldCat) was conducted up to 30^th^ September 2016. GoogleScholar, World Cat, ResearchGate, specific aquatic therapy websites and aquatic therapy journals were searched to identify additional literature. Full-text publications in English, German or Dutch were included. Studies were included when the intervention involved head-out cycling carried out in 10° to 35° Celsius water. Exclusion criteria were the use of wet suits or confounding interventions that would affect participants’ homeostasis. 63 articles were included and the study parameters of these studies were summarized. Using three grouping themes, included studies were categorised as 1) single session tests comparing aquatic versus land cycling, or 2) aquatic cycling only sessions investigating different exercise conditions and 3) aquatic cycling intervention programmes. Although the experimental conditions differed noticeably across the studies, shared characteristics were identified. Cardiovascular parameters were investigated by many of the studies with the results suggesting that the cardiac demand of aquatic cycling seems similar to land-based cycling. Only six studies evaluated the effect of aquatic cycling interventions. Therefore, future research should investigate the effects of aquatic cycling interventions, preferably in individuals that are expected to gain health benefits from aquatic cycling. Moreover, this comprehensive outline of available literature could serve as a starting point for systematic reviews or clinical studies on the effects of aquatic cycling on the cardiovascular responses.

## Introduction

Water-based fitness equipment has gained popularity within aquatic fitness leading to a development of dryland training machines, such as stationary exercise bikes and treadmills, into water-proof exercise gear. Although aquatic cycling has become a trending fitness activity, the modification of standard ergometer bicycles for aquatic programs is nothing new and stems from the late sixties. Researchers used water immersion as an effective simulation of prolonged weightlessness, moreover, the utilization of the aquatic environment has been recognized as useful in rehabilitation [1, 2]. Similar to land-based cycling, the repetitive circular movement of pedalling against the water resistance ensures a use of a large range of motion (ROM) of the lower limbs to improve cardiovascular fitness and muscle strength. The fact that individuals are sitting on the aquatic bike can be beneficial for those who have problems with balance and independent gait. However, in contrast, while the sitting position and hydrostatic pressure assist with postural control, the loss of free movement i.e. reduced challenges to balance, and the few variation of the exercises may limit its effect on functional capacity. A shared characteristic with other types of aquatic exercise is the decrease of joint loading due to the buoyancy of the water. During aquatic cycling participants are immersed in water up to the chest and the buoyancy of the water unloads the joints of the lower extremities and the lower spine, a condition appealing for patients experiencing pain or problems with physical functioning during exercising on land [3, 4]. Despite the potential benefits of aquatic cycling and its long history, the application of aquatic cycling in an exercise and clinical context still appears to be low. Limitations that might prevent clinicians using aquatic cycling for therapeutic purposes could include the investment costs, storage space requirements, and the elaborate set-up of the aquatic bikes. In particular, getting the bikes in and out of the pool, without an adjustable floor, is demanding.

The scientific evidence on the potential benefits of aquatic cycling seems to be scarce as well. Obvious search terms like aqua(tic) cycling, aqua(tic) bike or water cycling yield very few relevant results from scientific search engines. Moreover, the small number of references about aquatic cycling, used in previously published reviews on aquatic exercise, further emphasizes the impression of a scarcity of literature [5–7]. These reviews summarize the effects on head-out aquatic exercise, including aquatic cycling, or compared physiological responses of different types of aquatic exercise and swimming with each other [5–7]. Further, the aquatic cycling interventions were not described in detail in these prior reviews with these reviews only including cross-over studies.

Thus, the questions remain how has aquatic cycling been investigated in previous research, and whether a search effort solely on “aquatic cycling” would reveal additional publications and research investigating the effects of aquatic cycling intervention programmes. A systematic review with a meta-analysis would not suit this aim and therefore a scoping review study design was chosen. Systematic reviews are guided by specific research questions leading to strict in- and exclusion criteria. The primary aim for performing a scoping review is to map the available literature that meet a comprehensive research question combined without restricting inclusion criteria [8]. Where systematic reviews evolve out of an initial understanding of the research field, scoping reviews are employed to identify research and explore their features such as target populations, interventions, study designs and outcomes [8, 9]. As a result scoping reviews help to develop an understanding of the extent and possible gaps and uncertainties in the existing literature. Furthermore, a scoping review might identify a sufficient amount of studies that would facilitate a systematic review [9].

Therefore, the main objective of this study was to identify the scope of available research with regard to aquatic cycling as an exercise activity. Specifically, this scoping review aimed to explore the aquatic cycling exercises, study designs, comparison of training effects (if applicable), populations and outcomes utilised in research investigating aquatic cycling. To enable a comprehensive coverage of available literature the following research question was formulated: What is the available research on head-out aquatic cycling exercise?

## Methods

### Framework of a scoping review

The procedure of performing a scoping review follows similar steps as those used in systematic review approaches without limiting for study design of included studies and without a quantitative synthesis. The framework of Arksey and O’Malley for scoping reviews was implemented in this study [9]. The framework consists of five essential stages and one additional stage; 1) identifying the research question, 2) identifying relevant studies, 3) study selection, 4) charting the data, 5) collating, summarizing and reporting the results, and additionally 6) consultation of experts (optional). All stages can be performed in an iterative manner allowing refining of search parameters.

### Identifying relevant studies

A comprehensive literature search was conducted in August 2015 and updated to 30^th^ September 2016 in seven electronic academic databases (PubMed, MEDLINE, Cinahl, Embase, PEDro, Web of Science, WorldCat). The search strategy was documented by title of the database searched, date of the search, the complete search string that was used and the number of articles found (Table 1). The development of each search string was an iterative process and familiarisation with the literature revealed additional search terms for aquatic cycling such as “immersed cycling” or “underwater pedalling”. These terms were combined with more general terms for aquatic therapy (e.g. hydrotherapy) the search included the following key terms: ergometer, immersion, hydrotherapy, aqua(tic), cycling, underwater (bi)cycle ergometer, immersed ergocycle.

**Table 1:** Search strategy and results.

Database	Date	Search string	Results
PubMed	30-09-16	((ergometer[All Fields] AND (("immersion"[MeSH Terms] OR "immersion"[All Fields] OR "underwater"[All Fields] OR "aquatic"[All Fields]) OR ("hydrotherapy"[MeSH Terms] OR "hydrotherapy"[All Fields]))) OR ((aqua[All Fields] AND cycling[All Fields]) OR "underwater bicycle ergometer"[All Fields] OR "underwater cycle ergometer"[All Fields] OR "immersed ergocycle"[All Fields] OR "aquatic bike"[All Fields] OR "water bike"[All Fields])) AND "humans"[MeSH Terms]	120
MEDLINE	30-09-16	1. ((cycling and (hydrotherapy or aquatic exercise or aquatic therapy or water exercise or immersion)) or (aqua cycling or underwater bike or aquatic bike or immersed ergocycle or underwater bicycle ergometer or underwater cycle ergometer or underwater pedalling or underwater cycling or water bike)).af.	157
Cinahl	30-09-16	(TX ergometer AND ((aquatic therapy or hydrotherapy or aquatic exercise or water exercise) OR immersion)) OR underwater cycle ergometer OR immersed ergocycle OR aqua cycling OR underwater pedalling OR underwater bike OR aquatic bike OR water bike OR aqua bike)	30
Embase	30-09-16	1. ((cycling and (hydrotherapy or aquatic exercise or aquatic therapy or water exercise or immersion)) or (aqua cycling or underwater bike or aquatic bike or immersed ergocycle or underwater bicycle ergometer or underwater cycle ergometer or underwater pedalling or underwater cycling or water bike)).af.	194
PEDro	30-09-16	(ergometer AND immersion) (ergometer AND water exercise) (ergometer AND hydrotherapy) (aquatic bike) OR (aqua bike) OR (water bike) OR (underwater bike)	14
Web of Science	30-09-16	TS = (((ergometer AND (immersion OR hydrotherapy)) OR ((aqua AND cycling) OR underwater bicycle ergometer OR underwater cycle ergometer OR immersed ergocycle or aquatic bike or underwater pedaling or aqua bike or water bike))) Refined by: WEB OF SCIENCE CATEGORIES: (SPORT SCIENCES OR CLINICAL NEUROLOGY OR REHABILITATION OR PHYSIOLOGY OR MULTIDISCIPLINARY SCIENCES OR MEDICINE RESEARCH EXPERIMENTAL OR ENDOCRINOLOGY METABOLISM OR NEUROSCIENCES OR SURGERY OR RESPIRATORY SYSTEM OR PUBLIC ENVIRONMENTAL OCCUPATIONAL HEALTH OR MEDICINE GENERAL INTERNAL OR RHEUMATOLOGY OR ONCOLOGY OR ORTHOPEDICS)	145
WorldCat	30-09-16	ti:aqua cycling OR ((kw:immersion AND su:aqua-cycling) OR (ergometer AND hydrotherapy) OR (aqua bike) OR (aquatic bike) OR (water bike) OR (underwater bike))	5
**Total number of records**			**674**

af, all fields; TX, text; TS, topic; ti, title; kw, key word; su, subject.

Additionally, ResearchGate, GoogleScholar and relevant aquatic therapy websites (http://www.wcpt.org/apti, http://www.atri.org, https://www.aeawave.com) were examined. Moreover, the table of contents of the accessible key journals ‘International Journal of Aquatic Research and Education’ and ‘Journal of Aquatic Physical Therapy’ of the American Physical Therapy Association were checked for additional literature. Finally, reference lists of all included articles were hand-searched for new articles and the authors of this paper, all experts in the field of aquatic therapy and aquatic fitness, checked their own libraries for additional literature. The table of contents and reference lists were screened for the key words related to cycling and (immersion) exercise (testing) on land and in water. Throughout the search process it was noticed that no consistent terminology exists with regard to aquatic cycling. To ensure that the search terms used were correct and complete, the terminology used in included articles was re-evaluated. This post-hoc analysis (S1 File) addressing the terminology used to describe aquatic cycling confirmed our choice of search terms.

### Study selection

The inclusion and exclusion criteria were developed in two stages. In phase one, the authors agreed to include all formats of full-text reports that focused on the effects of head-out aquatic cycling exercise on the human body (Table 2, stage one). After familiarisation with the literature the selection criteria were further specified (Table 2, stage two). In each step of the selection procedure two or more reviewers were involved and inclusion discrepancies were solved by discussion. Screening of titles and abstracts was performed by two reviewers (BW and SR) with the online programme “Covidence” (Covidence systematic review software, Veritas Health Innovation, Melbourne, Australia, available at: www.covidence.org). Next, all authors were involved with the full-text screening and all results were independently imported into a Microsoft Excel file and compared after completion of the review process. Information on the two-stage development of the inclusion criteria is available in a supporting file (S2 File).

**Table 2:** Two stage expert consensus on inclusion and exclusion criteria.

**INCLUSION**
**Stage I**
• Full-text articles or master or doctoral theses written in English, Dutch, German
• Most of the following is described: intensity, duration of the session, body position on the bicycle, water temperature, and type of aquatic bike used
• Effect of head-out aquatic cycling on the human body is described
**Stage II**
• Participants have to be seated upright or semi-recumbent during immersed exercise
• The exercising limb has to be fully immersed in water
**EXCLUSION**
**Stage I**
• Full-body (above head) immersion of participants
• Use of self-contained underwater breathing apparatus (SCUBA)
**Stage II**
• Long duration resting immersion (>30 min) prior to exercise
• Confounding interventions that would affect participants homeostasis e.g. manipulation of participants’ glucose level or oxygen saturation
• Water temperatures below 10°C or above 41°C for resting immersion and water temperatures above 35.5°C for exercise conditions
• Use of wet-suits

### Charting the data

Descriptive data were extracted into Microsoft Excel tables including name of the first author, year of publication, primary research question, sample size, age, gender, health status of participants, exercise parameters, main results reported in the abstract, water temperatures, aquatic bike used and level of body immersion. Information on effects of resting immersion was not discussed for this review, but might have been part of the experimental set-up of the included studies. The tables were organised by the body position on the aquatic bike (upright and semi-recumbent), because physiological responses might vary with immersion level related to the body position on the ergometer [2]. All tables include information on interventions with healthy participants and patients. If patients were involved, information on the disease characteristics is reported in the tables. Articles that originated from the same data set, but focusing on different outcomes, were summarized and represented as one study in the tables, but references from all studies are included to aid identification of the separate articles.

## Results

**Fig 1:**
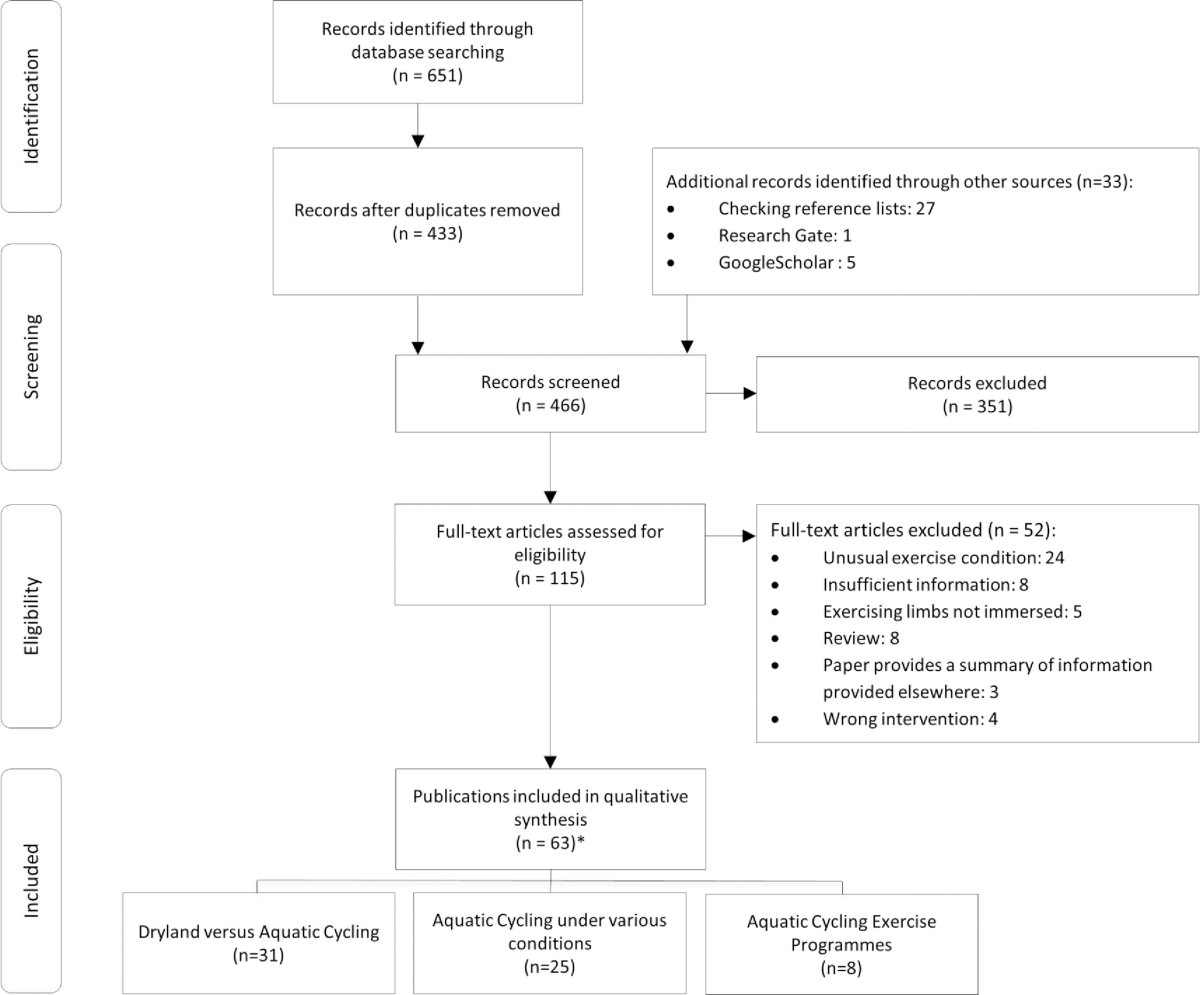
Flow diagram of identified publications. *One publication was allocated in two categories.

The search revealed 465 potential studies. After screening of the titles and abstracts, 350 studies were excluded and the full-text versions of 115 publications were read (Fig 1). Finally, 63 articles met the inclusion criteria. The reasons for exclusion during the full-text screening and the references of these excluded articles are presented in a supporting file (S3 File). Nevertheless, some of these publications might contain useful information and were therefore used as supportive literature. All included articles were published in peer-reviewed journals. Three of the included articles were published in German with an English abstract [10–12].

The included articles were categorized in three groups according to the intervention characteristics. The first group consisted of comparisons using the aquatic bike as a tool for evaluating land versus aquatic cycling. The second group consisted of studies on the physiological responses to single sessions of aquatic cycling under different exercise conditions (e.g. different water temperatures). Research on the effects of multiple aquatic cycling sessions was clustered in a third group. According to these three grouping themes the extracted data was organised in three tables (Table 2, Table 3 and Table 4).

**Table 3:** Land-based cycling versus aquatic cycling.

**UPRIGHT BODY POSITION**									
**Author**	**Year**	**Study design**	**Study aim**	**Sample**^**#**^	**Exercise parameters**	**Key findings**	**T**_**water**_	**Aquatic bike used**	**Immersion depths**
Garzon [16]	2016	Cross-over	To compare the early decay of HR recovery, a marker of parasympathetic reactivation, after a maximal incremental exercise on AC vs. LC	• n = 15 (F/M: 2/13) • age: 30±7	Land protocol: • Initial workload: 25W • Increments: 25W every min until exhaustion • Rpm: minimal 60 Water protocol: • Initial rpm: was set at 40 rpm (corresponding to P_ext_ of 25W) • Increments: 10 rpm until 70 rpm and thereafter by 5 rpm until exhaustion	• HR_max_ did not differ between AC and LC • More rapid* deceleration of HR in AC in the first minute of recovery • No difference in recovery HR in the late phase (minute 2–5 of recovery)	30°C	Hydrorider^®^	Chest
Wahl [41]	2016	RCT	To investigate the effect of AC vs. passive recovery on performance, muscle damage, muscle soreness and perceived physical state	• n = 20 M • age: 24.4±2.2	• Exercise: steady AC (vs. passive lying in supine position on land) after an strenuous exercise bout on land • Duration: 30min • Rpm: 65–75	• No differences between passive rest on land and AC with regard to performance, muscle damage and soreness and perceived physical state	31°C	Aquarider^®^	Chest
Sosner [42]	2016	RCT	To compare BP response after moderate LC, HIIT AC and HIIT LC	• n = 42 (F/M: 21/22) • BP > 130/85 mmHg • age: 65±10	Land protocol (moderate exercise): • 24min at 50% peak power output Land and water protocol (HIIT) • 6min warm-up at 50% peak power output, 2 sets of 10min: 15s 100% peak power output interspersed by 15s of passive recovery, 4 min passive (seated) recovery between sets	• HIIT LC and AC decreased 24hr BP* • HIIT AC modified 24-hour pulse-wave velocity	30°C	Hydrorider^®^	Chest
Garzon [14]	2015	Cross-over	To study the relationship between parameters of relative exercise intensity in AC and to establish a method for exercise intensity prescription in AC	• n = 33 (F/M: 5/28) • age: 33±10	Land protocol: • Initial workload: 25W • Increments: 25W every min until exhaustion • Rpm: minimal 60 Water protocol: • Initial rpm: was set at 40 rpm (corresponding to P_ext_ of 25W) • Increments: 10 rpm until 70 rpm and thereafter by 5 rpm until exhaustion	• Similar means of %HR_max_,%HR_reserve_ and %VO_2reserve_ for AC and LC • Predicted VO_2_ (L/min) = 0.000542 x rpm^2^–0.026 × rpm + 0.739 (*r* = 0.91, SEE = 0.319 L/min)	30°C	Hydrorider^®^	Xiphoid process
Garzon [13]	2015	Cross-over	To develop a mathematical model to calculate P_ext_ during AC with chest-level immersion for different pedalling rates and accounting for the drag forces exerted on the legs.	• n = 20 (F/M: 6/24) • age: 33±10	Land protocol: • Initial workload: 25W • Increments: 25W every min until exhaustion • Rpm: minimal 60 Water protocol: • Initial rpm: was set at 40 rpm (corresponding to P_ext_ of 25W) Increments: 10 rpm until 70 rpm and thereafter by 5 rpm until exhaustion	• P_ext_ (W) in water based on rpm = 0.0004 (rpm)^2.993^ (r^2^ = 0.99, SEE = 7.6 W, p < 0.0001) • When the P_ext_ was obtained on land, the rpm to generate an equal P_ex_ in water = 13.91 x DE P_ext_ (W)^0.329^ (r^2^ = 0.99, SEE = 1.5 W, p < 0.0001)	30°C	Hydrorider^®^	Xiphoid process
Garzon [15]	2015	Cross-over	To compare VO_2_, central hemodynamics and C(a-v)O2 during incremental maximal exercise and the subsequent hemodynamic recovery after AC and LC.	• n = 20 (F/M: 2/18) • age: 32±7	Land protocol: • Initial workload: 25W • Increments: 25W every min until exhaustion • Rpm: minimum of 60 Water protocol: • Initial rpm: was set at 40 rpm (corresponding to P_ex_ of 25W) • Increments: 10 rpm until 70 rpm and thereafter by 5 rpm until exhaustion	• At a comparable P_ext_ VO_2_ and C(a-v)O2 were lower** during AC • SV and Q were higher* during AC at comparable P_ext_ • During the recovery, VO_2_ and C(a-v)O2 remained lower** during AC while SV and ejection fraction were higher* in AC	30°C	Hydrorider^®^	Chest level
Yazigi [17]	2013	Cross-over	To compare cardiorespiratory response, BL, and thermal comfort during AC in neutral and warm water and LC	• n = 10 M • age: 22±1	Land protocol: • Initial workload: 75W • Increments: 35W every 3min until exhaustion • Rpm: 70 Water protocol: • Initial rpm: 50 rpm, • Increments: 10 rpm every 3min until 70 rpm and thereafter by 5 rpm every 3min until exhaustion	• HR_max_ and VO_2max_ were not sig. different in AC and LC • BL values were lower** in AC trials • VO_2_, HR, BL and thermal comfort scores were higher** at the end of the AC test compared to submaximal cadences in AC • Participants were more comfortable with AC in lower T_water_	27°C, 31°C	Hydrorider^®^	Xiphoid process
Finkelstein [27]	2011	Quasi-experiment	To compare BP and VO_2_ responses between pregnant and non-pregnant women, during AC and LC	• n = 20 F (10 non-pregnant F and n = 10 pregnant F at 27–29 wk gestation) • age: 31.9±3.1 (pregnant), 32.3±2.8 (non-pregnant)	Land + Water protocol: Series 1: • Initial workload: 25W • Increments: 25W every 2min until first ventilator threshold • Rpm: 50 Series 2: • 30min at the HR corresponding to the first ventilator threshold	• BP was lower** during AC in pregnant and non-pregnant F • No differences in VO_2_ between AC and LC and pregnant and non-pregnant F • After the first five-minute recovery period, both BP and VO_2_ were similar to pre-exercise values in pregnant and non-pregnant women, no difference between AC and LC	32.4°C	Sculptor–RGS, Brazil	Xiphoid process
Ferreira [40]	2011	Cross-over	To compare lactate removal during AC and passive recovery on land and in water	• n = 10 cyclists • age: 26.2 ±5.5	Land protocol: • Wingate Anaerobic Test on a ergometer Passive recovery (land + water): • 60min in supine position on land or in water (floating) Active recovery (water): • 30min of AC at up to 85% of the anaerobic threshold in water + 30min of sitting on the aqua bike	• After 15min the BL values were lower* in AC trials compared to passive recovery on land and in water • No difference between passive recovery on land and in water	28–30°C	Water Bike^®^	NR
Wiesner [18]	2010	Cross-over	To investigate the effect of water immersion on exercise-induced ANP release, lipid mobilization and lipid oxidation	• n = 17 M • age: 31±3.6	Land protocol: • Initial workload: 50W • Increments: 50W every 6min until exhaustion Water protocol: • Workload was increased by an increased number of fins to the flywheel • Rpm: NR	• HR, systolic BP and VO_2_ at the anaerobic threshold and during peak exercise were comparable in AC and LC • Respiratory quotient was lower* in AC • BL and glucose levels were lower* in water during peak AC • Free fatty acid concentrations were increased** with AC • Water immersion attenuated** (nor)epinephrine concentrations during peak exercise • ANP release was increased** in AC	28°C	Hydrobike Evolution^®^	Xiphoid process
DiMasi [39]	2007	Cross-over	To compare lactate removal during active recovery with AC or LC	• n = 11 M • age: 22.7±1.9	Land protocol: • Exercise bout on land treadmill: 2min warm-up, 6min at a speed 10% above the of the individual ventilatory threshold Land and water recovery protocol: • 15min AC or LC at 65% estimated HR_max_ (220-age)	• BL at 6 and 15 min of recovery was lower* in AC	30–31°C	Hydrorider^®^	Xiphoid process
Bréchat [33]	1999	Cross-over	To compare ventilator and metabolic requirements during AC and LC	• n = 15 M • age: 30±8, 29±8 (Series 1, 2)	Land and water protocol: • Series 1: subjects (n = 9), AC and LC at 60% VO_2max_ for 30min; Rpm: NR • Series 2: subjects (n = 9), AC and LC at workload of 122W for 30min; Rpm: NR	Series 1: • Ventilatory variables were comparable for both groups • Ergometric workload had to be reduced during AC to achieve exercise intensity of 60% VO_2max_ Series 2: • VO_2_ was higher** in AC • Min ventilation, tidal volume, respiratory frequency, and tidal inspiratory time were higher** in AC • BL was higher** in AC	33°C	EM designed in the authors laboratory	Xiphoid process
Hanna [28]	1993	Cross-over	To evaluate the effect of head-out water immersion on Q, SV and HR (at rest) and during graded submaximal AC and LC in men with a healed MI	• n = 15 M with history of MI • age: 49±3	Land protocol: • Initial workload: 40% of the subject’s VO_2peak_ • Increments: 25W every 6min until 75% of VO_2peak_ • RPM: 50 Water protocol: • Initial workload: 40% of the subject’s VO_2peak_ • Increments: increase in rpm (range 35–46) every 6min until 75% of VO_2peak_	• HR, Q and SV did not differ between AC and LC • No change in exercise response when patients with beta-blocker medication and exercise-induced ST-segment depression were excluded separately from the analysis	31°C	Modified Monark EM (Morlock& Dressen-dorfer)	Suprasternal notch
Sheldahl [19]	1992	Cross-over	To examine the influence of AC and LC on fluid-regulating hormones	• n = 10 M • age: 30±1	Land protocol: • Initial workload: 40% VO_2max_ • Increments: every 5min until exhaustion, resistance was adjusted to match 60, 80 and 100% of VO_2max_ • Rpm: 55–60 Water protocol: • Initial workload: 40% VO_2max_ • Increments: every 5min by an increase in rpm averaging: 39, 46, 52 and 59 rpm	• No group difference in VO_2Peak_ • Natriuretic peptide concentration was higher* in AC at 40% VO_2Peak_ and during recovery • Plasma renin activity was lower* in AC at 40% VO_2Peak_ and during recovery • Plasma aldosterone concentration was lower* in AC • Arginine vasopressin concentrations were lower* in AC • No group difference for osmolality and plasma sodium and potassium concentrations	32.5°C	Modified Monark EM (Morlock& Dressen-dorfer)	Shoulder
Katz [38], McMurray [37]	1990, 1993	Cross-over	To compare the effects of AC and LC on the mother and foetus	• n = 7 F at 25 wk gestation • age: NR	Land and water protocol: • Duration: 20min • Intensity: 70% VO_2max_, • RPM: predetermined according to Morlock & Dressendorfer	• Lower* HR and systolic BP during AC [38] • Higher* diuresis during AC [38] • Foetal HR showed a tendency to be higher after LC [38] • Lower T_rectal_ and T_mean body_ during AC compared to LC [37] • LC caused greater heat storage and sweat loss [37]	30°C	Modified Monark EM (Morlock& Dressen-dorfer)	Xiphoid process
Connelly [20]	1990	Cross-over	To compare the sympathoadrenal response to graded dynamic AC and LC	• n = 9 M • age: 22–36	Land protocol: • Initial workload: 40% VO_2max_ • Increments: every 5min until exhaustion, resistance was adjusted to match 60, 80 and 100% of VO_2max_ • Rpm: 55–60 Water protocol: • Initial workload: 40% VO_2max_ • Increments: every 5min by an increase in rpm averaging: 39, 46, 52 and 59 rpm	• Plasma norepinephrine concentration was reduced* at 80 and 100% of VO_2_ in AC • Plasma epinephrine and BL were similar in AC and LC at submaximal work stages, but both were reduced* AC at peak exertion • HR was lower* at 46, 52 and 59 rpm in AC • VO_2peak_ did not differ between AC and LC	32.5°C	Modified Monark EM (Morlock& Dressen-dorfer)	Shoulder
Christie [21]	1990	Cross-over	To compare cardiovascular responses during dynamic LC and AC exercise testing	• n = 10 M • age: 21–35	Land protocol: • Initial workload: 40% VO_2max_ • Increments: 3 increments of 6min, that matched 60, 80 and 100% of VO_2max_, workload increase was controlled by electronic resistance • Rpm: 55–60 Water protocol: • Initial workload: 40% VO_2max_ • Increments: 3 increments of 36–60 rpm every 6min, increments matched 60, 80 and 100% of VO_2max_	• VO_2max_ did not differ between AC and LC • Right arterial pressure, pulmonary arterial pressure, cardiac index, stroke index, left-ventricular end-diastolic and end-systolic volume indexes were higher* in AC • Arterial BP was comparable between groups • HR were lower* in AC at 80 and 100% VO_2max_	32,5°C	Modified Monark EM (Morlock& Dressen-dorfer)	Suprasternal notch
Mc Murray [32]	1988	Cross-over	To compare the cardiovascular responses during AC and LC in patients with coronary artery disease	• n = 10 M with coronary artery disease • age: 52	Land protocol: • Initial workload: 25W • Increments: 25W every 6min until completion of at least 3 increments • Rpm: NR Water protocol: • Initial workload: 30 rpm Increments: 10 rpm every 6min until completion of at least 3 increments	• Trend for HR to be less in AC during mild exercise • When matched for VO_2_, systolic BP were lower in AC • Q were slightly greater during AC than during LC, particularly at VO_2_ levels less than 1 l/min • Total peripheral resistance was greater* during LC	30°C	Modified Monark EM (Morlock& Dressen-dorfer)	Xiphoid process
Sheldahl [29]	1987	Cross-over	To assess the effects of central shift in blood volume on cardiorespiratory responses to dynamic AC and LC in middle-aged men	• n = 19 M • age: 48±8	Land and water protocol: • Initial workload: 35 to 40% of VO_2max_. • Increments: 150 kp-m every 6min until a work load that corresponded to 75 to 80% VO_2max_ • Rpm: NR	• Q was greater* in AC at 40 and 80% VO_2max_ • HR was lower* in AC at 80% VO_2max_ • Mean SV was greater* in AC at all exercise intensities	31°C	Modified Monark EM (Morlock& Dressen-dorfer)	Shoulder
Sheldahl [22]	1984	Cross-over	To investigate the effect of different levels of central blood volume on cardiac performance during submaximal exercise in supine and upright posture on land and in upright posture in water	• n = 12 M • age: 26.3±3.9	Land and water protocol: • Initial workload: 50W • Increments: 25W every 3min until exhaustion • Rpm: NR	• At submaximal workloads mean left ventricular end-diastolic /—systolic dimension were greater* in AC • At submaximal conditions HR did not differ between land and water trials • At a mean VO_2_ of 2.4 l/min, HR was greater** in the upright land posture than in upright posture in water • VO_2max_ did not differ between groups	31°C	Modified Monark EM (Morlock& Dressen-dorfer)	Shoulder
Dressen-dorfer [23]	1976	Cross-over	To determine the effect of head-out water immersion on cardiorespiratory responses to maximal aerobic work	• n = 7 M • age: 27	Land and water protocol: • Individual prescribed maximal workloads to achieve exhaustion within 4 to 5min	• HR, volume of expired gas per unit of time and maximum voluntary ventilation were lower* in AC • VO_2max_ did not differ between AC and LC	30°C	Modified Monark EM (Morlock& Dressen-dorfer)	Neck + chin
**SEMI-RECUMBENT BODY POSITION**									
**Author**	**Year**	**Study design**	**Study aim**	**Sample**^**#**^	**Exercise parameters**	**Key findings**	**T**_**water**_	**Aquatic bike used**	**Immersion depths**
Fenzl [24]	2015	Cross-over	To investigate changes in VO_2_—work rate relationship during increasing work rates in AC and LC	• n = 12 M • age: 35.1±5.4	Land and water protocol for arm-leg and leg exercise: • Initial workload: 50W • Increments: 25W every 2min until exhaustion • Contribution of arms during arm-leg exercise: 20% • Rpm: 70	• VO_2_ –work rate relationship is similar for arm-leg and leg exercise in AC and LC • Extra O_2_ cost by adding arm exercises is lower** with AC • At the ventilatory threshold two, exercise capacity, expressed as workload, is lower** in AC	27–28°C	Reha-Aquabike^®^	Xiphoid process
Fenzl [34]	2013	Cross-over	To compare the release of ANP and free fatty acids during prolonged AC with the release after an LC	• n = 6 M • overweight • age: 40.2±5.4	Land and water protocol: • 0-10min of testing protocol: adjustment of workload to reach a steady-state gas exchange at the anaerobic threshold. • 11-60min of testing protocol: cycling with set workload of moderate intensity • Rpm: NR	• ANP was higher** in AC • Free fatty acids were increased* post-exercise compared to baseline with no difference between AC and LC • Similar increase in epinephrine and decrease in insulin in AC and LC	27–28°C	Reha-Aquabike^®^	Xiphoid process
Fenzl [11]	2012	Cross-over	Comparison of gas exchange and the vagally modulated short time variability parameter to establish ventilatory threshold in water	• n = 12 M • age: 26–45	Land and water protocol: • Initial workload: 75W • Increments: 25W every 2min until exhaustion • Arm-leg workload ratio: 1:3 ratio • Rpm: 70	• The respiratory determined threshold heart rate is different* during AC and LC • Quantitative comparison of gas exchange measurements with HRV showed a strong correlation between both parameters	28°C	Reha-Aquabike^®^	Xiphoid process
Perini [25]	1998	Cross-over	To evaluate the effect of water immersion on the power spectrum of HRV (at rest) and during AC and LC	• n = 7 M • age: 22,0.9 (SEM)	Land protocol: Series1: • Initial workload: 0W • Increments: 20, 40, 60 rpm for 6min each Series 2: • Initial workload: 50W • Increments: 1 increment of 70W, followed by 50W increments for 6min each until exhaustion • Rpm: 60 Water protocol: - Series 1: Same protocol as above - Series 2: same as above except that the workload on the EM was set 25W below the values in LC	• The changes in power spectrum distribution of HRV occurring during exercise were similar in AC and LF • The central frequency of high frequency peak increased linearly with VO_2_, showing a tendency to be higher in AC at medium to high intensities	30°C	Modified Collins EM (Craig & Dvorak)	Chin level
Chen [26]	1996	Cross-over	To compare exercise tests with a semi-recumbent underwater exercise EM used on land and in water with a upright standard EM on land	• n = 10 (F/M: 3/7) • age: 30.6±6.5	Land and water protocol: • Initial workload: 0W • Increments: 44W (males) or 29W (females) every 2min until subjects could no longer maintain 60 rpm • Rpm: 60	• AC resulted in lower* total exercise duration, HR_max_, and maximal T_esophageal_ • The upright position in LC resulted in greater* total exercise duration and maximal power output than the semi-recumbent positions • VO_2max_ did not differ between positions and AC and LC	33°C	Modified Monark EM (Chen)	Clavicles
Israel [35]	1989	Cross-over	To determine a T_water_ that would attenuate the core rise that occurs with cardiovascular exercise	• n = 5 M • age: 26.8±4	Land and water protocol: • Workload: 60% of VO_2max_ for 30min in 21°C, 25°C and 29°C water and on land • Rpm: 50	• During exercise there was no change in T_rectal_ at water of 21°C and 25°C • T_rectal_ rose* during AC in 29°C water and during LC After recovery T_rectal_ is lower* for 21°C water and higher* for 29°C warm water compared to LC	21°C, 25°C, 29°C	Modified Collins EM (Craig & Dvorak)	Neck
Mc Ardle [36]	1984	Cross-over	To compare thermo-regulatory response to continuous exercise in different T_water_ and on land in males and females	• n = 18 (F/M: 8:10) • age: 23.1, range: 19–29	Land and water protocol: • Arm-leg EM exercise at 36W for 60min • Rpm: 30	• For men and women exercise at 1.7 l O_2_·min^-1^ prevented or retarded a decrease in T_rectal_ during AC • Similar thermoregulatory response were observed for men and women during exercise at each T_water_	20°C, 24°C, 28°C	Modified Collins EM (Craig & Dvorak)	1^st^ thoracic vertebra
Mc Ardle [30]	1976	Cross-over	To compare metabolic and cardiovascular adjustment to exercise on land and in different T_water_	• n = 6 M • age: 26±5.5	Land and water protocol: • Arm-leg EM workload: 0, 18, 36, 60, 84, 120W for 5min each • Rest: 10min between each workload • Rpm: 30	• During submaximal exercise in 18°C and 25°C water VO_2_ was higher* than in 33°C water • HR_max_ was lower* in 18°C and 25°C water than in 33°C water and during LC • Q–VO_2_ relationship was similar for AC and LC • At similar levels of VO_2_, SV was larger* in 18°C and 25°C water than in 33°C water and with LC	18°C, 25°C, 33°C	Modified Collins EM (Craig & Dvorak)	1^st^ thoracic vertebra
Craig [31]	1969	Cross-over	To compare cardiorespiratory responses during AC and LC	• n = 2 students • age: NR	Land and water protocol: • Workloads: 0, 18, 36, 60 and 84W • Duration workloads: 5,3,3, 1.5, 1.5min • Rpm: 30	• VO_2_ for a given workload was similar in LC and AC in 30°C and 35°C water • In 25°C water the VO_2_ averaged 0.14l/min more than in warmer water and with LC • Ventilation seemed somewhat greater in in cold water	25°C, 30°C, 35°C	Modified Collins EM (Craig & Dvorak)	1^st^ thoracic vertebra

AC, aquatic cycling; ANP, atrial natriuretic peptide; BL, blood lactate; BP, blood pressure; C(a-v)O2, arteriovenous difference; C, Celsius; EM, ergometer; F, female; HIIT, high-intensity interval training; HR, heart rate; HRV, heart rate variability; LC, land-based cycling; M, male; MI, myocardial infarction; min, minute(s); NR, not reported; P_ext_, external power output; Q, cardiac output; Rpm, revolution per minute; SE, Standard Error; SEM, standard error of mean; SV, stroke volume; T, temperature; VO_2_, oxygen uptake; W, Watts; wk, week(s); yrs, years

*, significant at p-value <0.05

**, significant at p-value <0.01

^#^If not stated otherwise participants are healthy and age is presented in years as mean±standard deviation.

**Table 4:** Aquatic cycling only (under various exercise conditions and in comparison to passive rest or immersion).

**UPRIGHT BODY POSITION**									
**Author**	**Year**	**Study design**	**Study aim**	**Sample**^**#**^	**Exercise parameters**	**Key findings**	**T**_**Water**_	**Aquatic bike used**	**Immersion depths**
Dionne [46]	2016	Quasi-experiment	To determine the effect of aquatic cycling and different levels of immersion on respiratory responses in healthy participants and people with a heart disease	• n = 34 participants (F/M: 10/24) • n = 21 (heart disease), n = 12 (controls) • age: 64.7±7.8 (heart disease), 61.0±7.8 (controls)	• Exercise: incremental, at different levels of body immersion • Initial rpm: 40 rpm • Increments: 10 rpm every 2min until at least one of the following was obtained: 85% of calculated HR_max_, a score of 16 on the Borg scale or an inability to reach and maintain cadence	• Immersion reduced ventilation in phase 1 of hyperpnoea by 79% at pedalling cadences of 40, 50 and 60 rpm in the heart disease group	29°C	Hydrorider^®^	Calf, hip, xiphoid process
Pinto[62]	2015	Cross-over	To compare the heart rate deflection point method with the ventilator method to determine the anaerobic threshold during AC	• n = 27 M • age:22.5±2.4	• Initial workload: 100 beats per min • Increments: 15 beat per min every 2min until exhaustion	• There was no difference between both methods for the determination of HR, %HR_max_, VO_2_, %VO_2max_ and cadence related to the anaerobic threshold	30°C	Hydrorider^®^	NR
Brasil [45]	2011	Cross-over	To investigate whether the type of exercise affects the physiological response to a AC and the perception of effort	• n = 10 F • age: 32.8±4.8	• Exercise: continuous and interval AC in seated and out-of-saddle-positions • Duration: 31min • Intensity: 75, 80, 85 and 92% VO_2max_ • Rpm: 80–100	• No differences between exercise protocols in HR, arterial BP, double product and BL concentration • Central RPE was higher** at 92%VO_2max_ of the continuous trial • Peripheral RPE was higher** at 85 and 92%VO_2max_ of the continuous trial	30°C	Hydrorider^®^	Xiphoid process
Giacomini [44]	2009	Cross-over	To assess HR and VO_2_ responses in men and women exercising on four different water EM	• n = 16 participants (F/M = 8/8) • age: 31.7±5.8	• Exercise: incremental exercise test on 4 aqua bike: with no resistance, with resistance added to the bottom bracket axle, with resistance added to the pedals • Initial rpm: 40 rpm • Increments: 5 rpm every 2min until the participant was unable to maintain the set rpm	• No difference in VO_peak_, HR_peak_ and rpm for gender • No difference in VO_peak_ and HR_peak_ between different bikes • Time to exhaustion and rpm at volitional exhaustion was different** across the 4 bikes • At 70 rpm the 4 aquatic bikes generated different** HR and VO_2_	25°C	4 different aqua bikes	Hips and thighs are immersed
White [51]	2005	Cross-over	To describe the acute effect of cold water temperature on post-exercise energy intake	• n = 11 M • age: 25.6±5	• Exercise: steady cycling • Duration: 45min • Intensity: 60% VO_2max_ • Rpm: NR	• Post-exercise energy intake was higher* after the cold water AC • Energy expenditure was similar for the cold and neutral water temperature trial	20°C, 33°C	Modified Monark EM (Morlock& Dressen-dorfer)	Mid-sternum
McMurray [52]	1994	Cross-over	To investigate the effects of anthropometrics and VO_2max_ on plasma cortisol and urine excretion of catecholamine and dopamine during exercise in different T_water_	• n = 11 M • age: 17–25 (range)	• Exercise: steady cycling vs. resting immersion • Duration: 30min • Intensity: 60% VO_2max_ • Rpm: NR	• Change in T_core_ was related** to T_water_ • Plasma cortisol increased ** from resting to exercise in 20°C water and decreased in 30°C and 35°C water • Dopamine and norepinephrine were higher during 20°C and 35°C* water AC exercise compared to exercise in 25°C and 30°C water • VO_2max_ and change in T_core_ were partial correlated* in 20°C water AC trial • BMI and change in T_core_ were partial correlated* during 35°C water trials	20°C, 25°C, 30°C, 35°C	Modified Monark EM (Morlock& Dressen-dorfer)	Neck
Katz [53, 54] McMurray [47, 55, 56]	1990 1988	Cross-over	To investigate the renal changes, foetal and uterine responses, thermoregulation, metabolic response and cardiovascular changes during immersion and AC in pregnant women	• n = 12 F at 15, 25 and 35 wk gestation and at 8 to 12 wk post-partum • age: 30±3(SE)	• Exercise: steady cycling • Duration: 20min • Intensity: 60% VO_2max_ • Rpm: NR	• Foetal HR were normal and unchanged from those at rest during exercise [54] • Post-exercise stress tests were reactive within 10min in 21 of 23 cases [54] • There was no uterine activity seen at 25 and 35 wk gestation [54] • Maternal serum alphafetoprotein was unaffected at all gestational ages [54] • T_maternal_ and calculated plasma volume did not change during exercise [54] • Diuresis was greater during pregnancy than postpartum, natriuresis was similar for all conditions [53] • T_rectal_, mean T_skin_, heat storage and evaporation during exercise and immersion in cold water were similar across different wk of pregnancy [56] • Compared to 10 weeks post-partum, pregnancy reduced heat storage, lowered T_skin_ and increased evaporative heat loss during immersion and exercise [56] • VO_2_ during AC was similar for all trials, but workload to achieve 60% VO_2max_ decreased* during 35^th^ wk of pregnancy [55] • Post-exercise BL declined with advancing pregnancy [55] • Blood glucose levels declined slightly* with exercise [55] • Blood triglyceride levels were elevated after AC compared to resting values at 25 wk gestation [55] • Plasma cortisol concentrations decreased with immersion and remained low during exercise [55] • Exercise HR was lower* in water than on land during pregnancy and post-partum [47] • Post-partum exercise cardiac output was lower* [47] • Post-partum total peripheral resistance was higher* [47]	30°C	Modified Monark EM (Morlock& Dressen-dorfer)	Xiphoid process
Shapiro [64]	1981	Cross-over	To modify a Monark ergo-meter applicable for AC graded exercise in which rpm could be maintained constant for prolonged periods of time	• n = 6 M • age: 25.8±2.1 (SE)	Series 1: • Exercise: AC with different combinations of 1 to 6 of fins and rpm’s • Duration: 1hr • Intensity: <85% VO_2max_ • Increments: 10 rpm every 12min • Rpm: Series 1:no fins = 15–63, one fin = 20–60, two fins = 20–50, three fins = 20–45, four to six fins = 20–40 Series 2: • Exercise: high intensity AC with different combination of fins and rpm’s • Duration: 1hr • Increments: 30, 40, 50, 60 rpm + different combinations of 1 to 6 fins	• 1 to 6 fins were attached to the flywheel to increase pedalling resistance • VO_2_ = (rpm)^b^ + 0.25, l·min^-1^, with a = 0.00164–0.00104n + 0.000266n^2^–0.00002n^3^; b = 1.64 + 0.506n–0.104n^2^ + 0.00667n^3^, when n is the number of fins • The correlation coefficient between measured and predicted VO_2_ was r = 0.98 • The preferable range of pedalling speeds was 29–40 rpm to maintain a constant speed for up to 1 h	26–29°C	Modified Monark EM (Shapiro)	Neck
McMurray [57]	1979	Cross-over	To compare the thermoregulatory responses of trained runners and swimmers to moderate AC in different T_water_	• n = 11 M athletes • age: 20.8±1.1 (SE) (n = 5 runners), 18.5±0.5(SE) (n = 6 swimmers)	• Exercise: steady AC • Duration: 30min • Intensity: 60% VO_2max_ • Rpm: NR	• Changes in metabolic rate were greater* for runners in 20°C water and for swimmers in 30°C and 35°C water • Runners had higher* sweat rates during exercise in 35°C water • Swimmers thermoregulated better in 20°C water than runners	20°C, 25°C, 30°C, 35°C	Modified Monark EM (Morlock& Dressen-dorfer)	Neck
Dressen-dorfer [23]	1976	Cross-over	To determine the effect of water temperature on VO_2max_ and HR_max_	• n = 4 M • age: 26	• Individual prescribed maximal workloads to achieve exhaustion within 4 to 5min	• T_water_ had no significant effect on VO_2max_ • HR was 8 and 15 beats per min lower in 30°C and 25°C water compared to 35°C water	25°C, 30°C, 35°C	Modified Monark EM (Morlock& Dressen-dorfer)	Neck + chin
Morlock [63]	1974	Cross-over	To modify a standard land EM for underwater use and to measure VO_2_ as a function of rpm	• n = 6 M • age: 24–29 (range)	• Exercise: incremental exercise • Increments: 5min at 20, 40, 50, 60 and maximal rpm	• Modifications: installation of 2 grease nipples for regreasing l, installation of a magnetic reed switch to monitor rpm, removal of the friction belt • VO_2_ = 0.274+0.000008rpm^3^, r = 0.996	30°C	Modified Monark EM (Morlock& Dressen-dorfer)	Neck
**SEMI-RECUMBENT BODY POSITION**									
**Author**	**Year**	**Study design**	**Study aim**	**Sample**^**#**^	**Exercise parameters**	**Key findings**	**T**_**water**_	**Aquatic bike used**	**Immersion depths**
Fujimoto [48]	2016	Cross-over	To investigate the effects of T_water_ on cardiorespiratory responses and exercise per- formance	• n = 10 M • age: 22±2	• Exercise: incremental exercise • Increments: initial workload 60W, increased by 20W every 2min for the first four levels and then by 10W every minute until exhaustion • Rpm: 60	• VO_2peak_ did not differ between T_water_ • At submaximal intensities (60–120 W), VO_2_ was greater* at T_w_ = 18°C than at 26 or 34°C • Max. workload was lower* at T_w_ = 18°C than at 26 or 34°C	18°C, 26°C, 34°C	Aerobike 330 Combi	Shoulders
Fenzl [12]	2010	Cross-over	To compare the effects of different T_Water_ on BP, HR and pressure frequency product	• n = 8 M • age: 25–49 (range)	• Exercise: 3 trials in 26°C, 32°C and 35°C water • Duration: 10min per T_water_ and intensity • Intensity: 26, 41, 52% VO_2max_ Rpm: NR	• HR was highest* during AC in 35° water • Pressure frequency product was higher* during AC in 35°C compared to AC in 26°C water • Systolic BP was similar in all conditions	26°C, 32°C, 35°C	?	Hand-breadth above xiphoid process
McArdle [43]	1992	Cross-over	To compare the influence of exercise intensity on thermoregulation in men and women in cool and cold water	• n = 16 (F/M: 8/8) • age: 23.3, range: 19–29	• Exercise: steady AC vs. resting immersion • Duration: 1hr • Intensity: Level I = 700ml O_2_·min-^1^, Level II = 1250ml O_2_·min-^1^, Level III = 1700ml O_2_·min-^1^ • Rpm: NR (arm-leg exercise was performed at the same rate of limb movement)	• For men and women of similar body fat %, decreases in T_rectal_ were greater* for women during resting immersion and level I exercise in 20°C water • With level II and III exercise in 20°C water women maintained a 0.2°C higher T_rectal_	20°C, 28°C	Modified Collins EM (Craig & Dvorak)	1^st^ thoracic vertebra
Sogabe [65]	1987	Cross-over	To describe a simple modification of a conventional row-bicycle EM applicable for graded horizontal exercise in water	• n = 7 M • age: 34.4±2.9 (SE)	• Exercise: exercise tests with various combinations of pedalling speeds and size of fins • Initial rpm: 20 • Increments: 10 rpm every 10min until 69 (no fins) to 60 (large fins)	• EM modifications: replacement of the saddle with a plastic seat, fastening of fins to the pedal cranks, removal of the handle • Preferred rpm for a prolonged time: 30–40 • Workload that was achieved by the attachment of the fins: VO_2_ of 400–2000 ml/min	31°C	Modified row-cycle EM (Sogabe)	Neck
Golden [58]	1987	Cross-over	To describe the thermal response of leg exercise compared to static immersion in cold water	n = 15 M age: 14–34 (range)	• Exercise: steady AC vs. resting immersion • Duration: 40min • Intensity: VO_2max_ in litres /0.05 • Rpm: 60	• T_rectal_ and T_aural_ showed a greater** decrease with static immersion compared to exercise between 10 and 30 min of the trial	15°C	Modified electroni-cally braked Siemens bicycle EM	NR
Toner [59]	1986	Cross-over	To investigate the role of morphology and body mass on thermal and metabolic responses to AC	• n = 10 M (n = 5 large body mass, n = 5 small body mass) • age: NR	• Exercise: steady cycling (vs. resting immersion) • Duration: 1hr • Intensity: VO_2_ of 1.5 l·min^-1^ • Rpm: NR	• Metabolic rate, T_rectal_ and T_esophageal_ were not different between the small body mass and large body mass group during AC	26°C	Modified Monark EM (Shapiro)	Neck
Toner [49]	1986	Cross-over	To examine the relationship between physiological and perceptual variables over time and across water temperature during various modes of AC.	• n = 8 M • age: 22.4±3.6	Exercise: leg vs. arm-leg vs. arm exercise • Duration: 45min • Intensity: high (60% VO_2peak,_ VO_2peak_ matched across T_water_) vs. low (40% VO_2peak,_ power output matched across T_water_) • Rpm: 40	• VO_2peak_ did not differ between types of exercise and T_water_ • RPE during low intensity exercise did not differ between T_water_ • RPE during high-intense exercise was lower for 20°C water • RPE was moderately correlated with HR (r = 0.68) and ventilation (r = 0.61)	20°C, 26°C,	Modified Monark EM (Shapiro)	Neck
Toner [60]	1985	Cross-over	To compare the thermal and metabolic response during resting immersion and AC in cool and cold water	• n = 9 M • age: 23.6±5.2	• Exercise: steady cycling (vs. resting immersion) • Duration: 1hr Intensity: VO_2_ of 1.6 l·min^-1^ • Rpm: NR	• Metabolic rate, T_skin_, T_rectal_ and T_esophageal_ were higher* during AC compared to resting immersion • Heat flows were greater* during AC than with resting immersion	18/20°C, 30°C	Modified Monark EM (Shapiro)	Neck
Toner [61]	1984	Cross-over	Thermal and metabolic response during arm, leg and combined arm-leg exercise	• n = 8 M • age: 22.4±3.6	• Exercise: leg vs. arm-leg vs. arm exercise • Duration: 45min • Intensity: high (60% VO_2peak_) vs. low (40% VO_2peak_) for leg and arm-leg exercises, only low intensity for arm exercises • Rpm: 40	• In all T_water_ there was no difference between exercise types in final metabolic rate during low intensity • Final T_rectal_ during low intensity exercise for arm, arm-leg trials were lower* than for leg trials • At high intensities finale T_rectal_ were lower* for arm-leg than for leg exercise in all T_water_ • No difference between exercises in final T_skin_ and heat flow values • Metabolic rate was lower in leg exercise compared to arm-leg exercise at high intensity in 20°C water	20°C, 26°C, 33°C	Modified Monark EM (Shapiro)	Neck
Craig [50]	1968	Cross-over	To investigate thermal regulation during heavy and light exercise in cool and warm water	• n = 10 M • age: 27±5.8	• Exercise: steady AC • Duration: 60min • Intensity: light workload (VO_2_ = 0.70 litres/min) vs. heavy workload (VO_2_ = 0.92 litres/min). Workload was increased by an increase in pedalling resistance • Rpm: 30	• VO_2_ was higher during the last 30 min of light exercise in 24°C • After an initial increase in T_ear_, T_ear_ decreased with light exercise in water with ≤ 32°C and with heavy exercise in 24°C water • T_rectal_ continuously declined with light work load and in water with ≤ 32°C, when exercising with heavy workload an initial decrease of rectal temperature was followed by and increase that persisted with T_water_ of 28–32°C	24–35°C	Modified arm-leg EM (Craig & Dvorak)	Neck

AC, aquatic cycling; BL, blood lactate; BMI, body mass index; BP, blood pressure; C, Celsius; EM, ergometer; F, female; HR, heart rate; HRV, heart rate variability; LC, land-based cycling; M, male; min, minute(s); NR, not reported RPE, rate of perceived exertion; Rpm, revolution per minute; SE, Standard Error; T, temperature; VO_2_, oxygen uptake; wk, week(s)

*, significant at p-value <0.05

**, significant at p-value <0.01

^#^If not stated otherwise participants are healthy and age is presented in years as mean±standard deviation.

### Land-based cycling compared to aquatic cycling

Thirty-one studies compared aquatic cycling with land cycling (Table 3). Half of the studies (n = 15) used a maximal incremental exercise test to investigate the physiological responses during immersion versus on land exercise testing [11, 13–26]. Submaximal incremental exercise tests were conducted in six studies [27–32]. Increments were mostly achieved by an increase in pedalling frequency. Seven studies of the aforementioned studies controlled exercise intensity by electronically regulated pedalling resistance [10, 21, 24–27, 31]. An additional six studies compared submaximal continuous aquatic cycling with land cycling [33–38]. Three other studies evaluated aquatic cycling as a mean for active recovery after an extensive exercise bout on land [39–41]. Furthermore, one study compared the effect of moderate intense dryland cycling with high-intensity interval training (HIIT) on land and in water [42]. Two-third of the aquatic cycling sessions (n = 22) were conducted in an upright body position. Nine studies [11, 24–26, 30, 31, 34–36] compared semi-recumbent cycling on land and in water. Four semi-recumbent bikes also had arm pedals [11, 24, 30, 36]. The level of body immersion of the participants varied from chest level to chin level. The water temperature during the exercise sessions ranged from 18°C to 35°C.

All but three studies used a cross-over design to compare both environments. Additional study designs were a randomized controlled trial [41, 42] and a quasi-experimental study [27]. In 19 out of 31 studies participants were young, healthy males. Five studies included healthy participants of both sexes [13–16, 26, 36] and three studies included pregnant women [27, 37, 38]. In four other studies participants were middle-aged men [29], males with cardiovascular diseases [28, 32] and men and women with hypertension [42].

Studies (n = 21) investigating the difference in cardiovascular responses between aquatic versus land cycling compared oxygen consumption (VO_2_), heart rate (HR), stroke volume, cardiac output and blood pressure [15–23, 26–33, 37, 38, 42]. In total eight studies investigated the maximum VO_2_ response during land and aquatic cycling, with all but one study [15] reporting equivalent VO2_max_ values achieved by the participants on land and in water [17–23, 26]. Maximal HR was found to be lower during aquatic cycling at intensities higher than approximately 80% of the VO_2max_ in seven from ten studies [20–23, 26, 29, 30]. The remaining three studies reported similar maximal HR for the land and water conditions [16–18]. In men, following recovery from a myocardial infarction, no difference in submaximal HR on land and in water was found [28]. McMurray et al. reported a trend toward a lower HR at submaximal intensities in water in men with coronary heart disease [32]. In pregnant women moderate aquatic cycling resulted in lower maternal and foetal HR compared to land-based cycling [38]. Four studies reported higher stroke volume and cardiac output in the aquatic cycling group consiting of healthy participants [15, 21, 29, 30]. Systolic blood pressure was similar in healthy males during an incremental exercise test when using aquatic versus land-based cycling [18, 21]. In pregnant women and in men with coronary artery disease the systolic blood pressure was reported to be lower during submaximal aquatic cycling [27, 32, 37, 38]. Sosner et al. reported a similar post-exercise reduction in blood pressure in patients with hypertension after a high-intensity cycling session on land and in water [42].

Other key outcomes were ventilation parameters [23, 31, 33], lipid mobilisation and oxidation [18, 34], sympathoadrenal response [18, 20, 34], lactate accumulation and removal [17, 18, 20, 39, 40]. and thermoregulatory responses [35–37]. Further outcomes were the development of prediction equations to estimate oxygen consumption from pedalling rate during aquatic cycling [13, 14] and to calculate external power output of aquatic cycling [13]. Fenzl et al. compared the gas exchange measurements with the heart rate variability to estimate the ventilator threshold on an arm-leg aquatic bike [11].

### Aquatic cycling under different exercise conditions

Twenty-five studies investigated the effect of several different exercise conditions during aquatic cycling (Table 4). The comparisons are based on cross-over studies with healthy young males with the exception that healthy (non-pregnant) females were included in three studies [43–45] and one study used a quasi-experimental design to compare age-matched healthy controls with heart disease patients [46]. Common core outcomes were cardiovascular [12, 23, 44, 45, 47–50], metabolic [36, 51–55] and thermal response [43, 49, 50, 52, 56–61] to different exercise conditions. Furthermore, approaches to estimate and regulate exercise intensity during aquatic cycling were evaluated [62–64].

Different exercise conditions were created mostly by changes in water temperature [12, 23, 43, 48–52, 57, 60, 61] and different exercise intensities (high versus low) [23, 43, 44, 50, 58–61, 63–65]. With regard to the exercise parameters intensity and duration, studies (n = 11) utilised continuous, submaximal exercise (40 and 60% of VO_2max_) with a duration of 30 to 60 minutes [12, 23, 43, 49, 51, 52, 57, 60, 61]. Exercise intensities were either based on graded exercise testing on land [12, 23, 44, 45, 47, 50, 51, 53–56, 58] or in water [23, 43, 46, 48, 52, 57, 62–64]. The water temperatures that were compared ranged from cold (18–20°C) and cool (25°C) to thermoneutral (30–35°C). Other studies compared different levels of body immersion [46], different types of exercise (interval versus continuous cycling, arm versus arm-leg versus leg exercise) [45, 49, 61] and different aquatic bikes with each other [44]. Furthermore, the maternal and foetal response to submaximal (60% of VO_2max_) aquatic cycling during different stages of pregnancy was studied [47, 53–56].

Fifteen studies used upright aquatic bikes [23, 44–47, 51–57, 62–64]. In all these studies pedalling frequency regulated exercise intensity while two studies focused on the influence of pedalling resistance provided by additional fins to the flywheel [44, 64]. Sogabe et al. used the additional fins to increase pedalling resistance in semi-recumbent cycling [65]. In all other semi-recumbent bikes intensity was set with electronically controlled pedalling resistance mechanisms [43, 48–50, 58–61].

### Aquatic cycling intervention programmes

In total eight intervention studies, investigating the effects of a multiple sessions aquatic cycling exercise programme, were found [66–73]. The exercise programmes (Table 5) lasted between three and 36 weeks with an exercise frequency between two and five times per week. The duration of one session varied between 30 and 90 minutes. Exercise intensities were based on land-based maximal graded exercise-tests and the training intensities were set between 60 and 80% of the VO_2max_ in all but one study [66]. In a one-group test-retest study, Sheldahl et al. assessed weight loss in obese women after a low intense (30 to 40% of VO_2max_) aquatic cycling programme [66]. Boidin et al. also evaluated the effects of aquatic cycling on cardiometabolic parameters in obese people [71]. In this retrospective study the participants underwent an extensive lifestyle programme including high-intensity aquatic cycling or land cycling. Furthermore, two randomised studies evaluated the cardiovascular effect of aquatic cycling compared to land cycling in young healthy males [68] and patients with multiple sclerosis [72, 73]. Two quasi-experimental studies investigated the influence of water temperature on heat tolerance and aerobic capacity [67, 69, 70].

**Table 5:** Aquatic cycling intervention programmes.

UPRIGHT BODY POSITION									
Author	Year	Study design	Study aim	Sample^#^	Exercise parameters	Key findings	T_Water_	Aquatic bike used	Immersion depths
Boidin [71]	2015	Retro-spective cohort: AC vs. LC	To compare the effects of a lifestyle intervention in addition to AC or LC on cardiometabolic and exercise parameters in obese patients	n = 95 obese people • AC: n = 21 (F/M: 19/2) age: 58±9 • LC: n = 74 (F/M: 55/19) age: 55±7	• Exercise programme: HIIT AC + water-based resistance training, 5x Mediterranean diet counselling • Programme duration: 36 wk • Frequency: 2-3/wk • Duration session: 34min AC, 20min resistance exercise • Intensity:15 RPE/ 80% MAP • Rpm: NR	• Reduction** in body mass, WC, total and trunk fat mass; no group difference • Improvement* in resting BP, maximal aerobic capacity, resting HR; no group difference • Improvement* in fasting glycaemia, triglyceride levels; no group difference • Improvement* in abdominal and thigh muscle endurance; no group difference	NR	Hydrorider^®^	NR
Bansi [72, 73]	2013	RCT: AC vs. LC	To investigate the influence of exercise in cytokine response, health-related QoL, fatigue, neurothophin concentrations and cardiorespiratory values	n = 60 MS patients • AC: n = 25 (F/M: 17/8), age: 50, range: 44.6–55.1 • LC: n = 28 (F/M: 18/10), age: 52, range: 46.7–56.3	• Exercise programme: steady AC + usual care rehabilitation • Programme duration: 3 wk • Frequency: 5/wk • Duration session: 30min • Intensity: lactate threshold = 60% VO_2peak_ • Rpm: 50–60	• Short term immune adaptions and increased VO_2_ lactate values were associated with improved health-related QoL and reduced fatigue [73] • Improved health-related QoL; no group difference [73] • Improved self-reported physical fatigue; no group difference [73] • Cytokines and neurotrophins showed no change over time and between groups [72] • Fatigue scores associated with baseline and post-intervention exercise tests remained unchanged in both groups [72] • Cardiorespiratory values improved** over time; no group difference [72]	28°C	Aquarider Professional^®^	1,30m
Young [69, 70]	1995, 1993	Quasi experi-ment: hot vs. cold water	Comparison of metabolic and thermal adaption to endurance training in hot and cold water and its effect on aerobic capacity	n = 18 M • 20°C water: n = 9, age: 20±1 • 35°C water: n = 9, age: 20±1	• Exercise programme: steady AC in hot or cold water • Programme duration: 8wk • Frequency: 5/wk • Duration session: 60min • Intensity: 60%VO_2max_ • Rpm: 40	• Reduced* post-exercise muscle glycogen use; no group difference [70] • Lactate accumulation was equal for hot and cold water [70] • 13% post-intervention increase** of VO_2max_; no group difference [69, 70] • 4% increase** of erythrocyte volume; no group difference [69] • Unchanged plasma volume; no group difference [69] • 38% increase** of vastus lateralis citrate synthase activity; no group difference [69]	35°C vs. 20°C	Modified Monark EM (Shapiro)	neck
Avellini [67]	1982	Quasi experi-ment: warm vs. cold water vs. land	>To determine how physical training on land compared to warm and cold water training affects heat tolerance	>n = 15 M • land: n = 5, age: 23.2± 4.7 • 32°C water: n = 5, age: 20.8±1.8 • 20°C water: n = 5, age: 23.0±4.1	>• Exercise programme: steady AC in warm and cold water. Groups were divided based on the maximal exercise capacity, body surface area, and % body fat. • Programme duration: 4wk Frequency: 5/wk • Duration session: 60min • Intensity: 75%VO_2max_ • Rpm: NR	>• Similar increase in VO_2max_; no group difference • Higher* post-training values of T_rectal_ for LC compared to AC • 0.9°C reduction* of post-training mean T_skin_ for LC and warm water AC. • Post- training total body sweat: increased* only warm water AC (25%). • Post-heat acclimation: only cold water AC demonstrated an increase* in sweat rate (25%) • Decrease* in post-training HR in all 3 groups, greatest decline (29 beats·min) in land group. HR in warm and cold-water AC HR decreased 14 and 18 beats·min. • Post-heat acclimation HR: reduction* from post-training values in all groups	>20°C vs. 32°C	>Modified Monark EM	>neck
Sheldahl [68]	1986	RCT: AC vs. LC vs. control	Comparison of land and water training to determine whether the cephalad shift in blood volume due to water immersion affects normal adaptations to aerobic endurance training	n = 22 M • • age: 49±8 • water: n = 9 • land: n = 9 • control: n = 4	• Exercise programme: steady AC Programme duration: 12wk • Frequency: 3/wk • Duration session: 30min • Intensity: 60–80% VO_2max_ • Rpm: NR	• Increase* in SV at submaximal exercise intensities; no difference between AC and LC • Increase** in VO_2max_ in both exercise groups • Decrease** in HR at submaximal exercise intensities; no difference between AC and LC • Decrease* in BP at submaximal exercise intensities in LC and AC group	31°C	Modified Monark EM (Morlock & Dressen-dorfer)	Shoulder
Sheldahl [66]	1982	Single group test- retest	To investigate if AC in cold water leads to weight loss	n = 7 obese F age: 31.4±11.1	• Exercise programme: steady AC in cold water. T_water_ was determined in preliminary tests in 31°C, 30°C, 28°C, 24°C and 20°C water. • Programme duration: 8wk • Frequency: 5/wk • Duration session: 90min • Intensity: 30–40%VO_2max_ • Rpm: NR	• No change in body weight, body fat, fat-free body weight • Constant caloric intake throughout the intervention • VO_2max_ did not change	17–22°C	Modified Monark EM (Morlock & Dressen-dorfer)	neck

AC, aquatic cycling; BL, blood lactate; BP, blood pressure; C, Celsius; EM, ergometer; F, female; HIIT, high-intensity interval training; HR, heart rate; LC, land-based cycling; M, male; m, meter; min, minute(s); MS, multiple sclerosis; NR, not reported; QoL, quality of life; RCT, randomized controlled trial; Rpm, revolution per minute; SV, stroke volume; T, temperature; VO_2_, oxygen uptake; W, Watts; WC, waist circumference; wk, week(s)

*, significant at p-value <0.05

**, significant at p-value <0.01

^#^If not stated otherwise participants are healthy and age is presented in years as mean±standard deviation.

Four studies reported a significant improvement of cardiorespiratory parameters compared to baseline in healthy (obese) people and multiple sclerosis patients [68, 71–73]. Aquatic and land cycling evoked similar improvements in cardiorespiratory parameters. Further, moderate land and aquatic cycling achieved similar improvements in health-related quality of life and self-reported physical fatigue in patients with multiple sclerosis [72, 73]. Boidin et al. reported comparable results in weight loss and reduction in fasting glycaemia and triglyceride levels in obese people [71]. In obese women, an eight week aquatic cycling programme in cold water did not lead to weight loss [66].

In young, healthy males, there was no superior effect of cold or warm water on the improvements in cardiovascular parameters [67, 69, 70], lactate accumulation lactate accumulation [69], dryland heat tolerance [67] and muscle glycogen utilization [69]

## Discussion

This is the first review to scope the available literature on head-out aquatic cycling exercise. The aim of this review was to describe the study parameters of available research utilising aquatic cycling as an exercise modality. Sixty-three publications were identified and the review provides a full summary of the set-up of aquatic interventions and possible comparisons, core outcomes, involved participants and the study designs utilised in current literature. The exploration of the intervention parameters revealed great variety on the use and execution of aquatic cycling.

### Land-based cycling versus aquatic cycling

The main body of the current research on aquatic cycling focuses on cardiovascular outcomes and the core findings for the comparison between land-based and water-based cycling showed similar trends. These latter studies [17–23, 26] reported comparable VO_2max_ values of aquatic and land-based cycling and therefore, the cardiac demand of aquatic cycling seems similar to land-based cycling. The results for HR were less consistent with a tendency for a lower HR during aquatic cycling compared to land-based cycling [20–23, 26, 29, 30]. Further, cardiac output and stroke volume was reported to be higher during aquatic cycling [15, 21, 29, 30]. These results are in line with the general understanding concerning the effects of water immersion on the human body. Hydrostatic pressure exerts external pressure on the immersed body, which increases with increased depth [2, 74]. Due to the hydrostatic pressure exerted there is a shift of blood from the extremities to the chest cavity, increasing arterial filling, and thus cardiac output and stroke volume are increased [2, 74]. Because cardiovascular parameters are modified by immersion, this could explain why the literature is inconclusive on the optimal recommendations for exercise prescription during aquatic cycling. Another explanation maybe as most aquatic bikes are not equipped with an electronically controlled pedalling resistance mechanism and approaches to estimate VO_2_ from aquatic cycling are often based on pedalling frequency, with or without additional resistance. However, these equations cannot be used for all aquatic bikes, as the design and drag resistance created by pedals and resistance fins vary considerably across the aquatic bikes.

### Aquatic cycling under different conditions

Due to the heterogeneous nature of aquatic cycling, many variables are involved when studying its impact on individuals, for example device-specific factors [44, 63–65] or environmental parameters as water temperature [12, 23, 43, 48, 49, 51, 52, 56–61]. Thus explaining why the cardiovascular response to different exercise conditions was frequently investigated. For example, it seems that VO_2max_ is comparable across different water temperatures and that participants perceived exercising in warm water as more exhaustive [23, 48, 49]. Further, included studies concluded that exercise intensities up to maximal limits are achieved by an increase in pedalling frequency and that VO_2peak_ does not differ between the different types of aquatic bikes [44, 64]. However, high-pedal frequencies are difficult to maintain during longer exercise sessions with a continuous character [44, 64]. To avoid discomfort with maintaining high pedal frequencies, exercise intensity can be modified by an increase in pedalling resistance or by utilising an interval training [45]. The latter was perceived less exhaustive than a continuous protocol [45].

### Aquatic cycling as an intervention

Only six studies investigated the effect of multiple aquatic cycling sessions [66–73]. In four studies aquatic cycling was used in a clinical context for patients with multiple sclerosis and as exercise training for older adults and obese individuals. Research showed that aquatic cycling was equally effective than land-based cycling for improving cardiovascular fitness [66, 68, 71–73]. Furthermore, none of the included studies reported adverse events related to the training, suggesting that aquatic cycling is a safe exercise modality.

Most of the exercise protocols of the aquatic cycling intervention programmes consisted of steady cycling in a seated position with moderate intensity. Only Boidin et al. used an interval protocol for the training of obese individuals [71]. It seems that the full potential of aquatic cycling including out-of-saddle positions and arm and trunk exercises is not published yet in peer-reviewed journals [7]. Addition of these elements might prevent monotony especially in multiple session programmes [75] and results from supportive literature suggest that a full spectrum aquatic cycling programme is effective in patients with musculoskeletal disorders [76].

This scoping review has identified a number of areas for further research. Most of the included studies have a cross-over design with few cycling sessions and investigated the exercise response in young healthy males, because gender, body mass and morphology are known to affect the response to aquatic cycling [59, 77, 78]. Further, only six studies investigated the effect of an aquatic cycling intervention programme. To improve the use of aquatic cycling in healthcare, future studies, preferably RCTs, should investigate the effects of aquatic cycling interventions in different populations and on outcomes such as (joint) pain, muscle strength or physical functioning, which are yet to be investigated. Of specific interest may also be the biomechanics of aquatic cycling and differences of seated and out-of saddle cycling. Furthermore, the identified literature seems suitable for more systematic reviews. For example it seems worthy to synthesize the available evidence on cardiovascular responses to aquatic cycling.

To further improve the understanding of acute and long-term physiological adaptions to aquatic cycling training and facilitate between study comparisons, consistent reporting of the following parameters is recommended. Studies should describe the type of aquatic bike, body position, level of immersion, water temperature, methods used to control and assess exercise intensity i.e. training frequency, duration, rpm and pedalling resistance. Furthermore, it should be stated whether or not adverse events occurred. In addition to an accurate description of the aquatic cycling intervention, an agreement of experts on uniform keywords to describe the exercise activity is also strongly advised since this would improve the search in scientific databases. In this review the terms “aquatic cycling” and “aquatic bike” were used, as these expressions nowadays are commonly associated with this type of exercise.

This review has strengths and weaknesses. The extensive search procedure in this review resulted in more than sixty publications on aquatic cycling only, which were summarized and displayed. However, the presented studies should be interpreted with caution, because no quality assessment of the internal validity of the included studies was made in order to cover a broad spectrum of literature. Furthermore, this review provides a very general overview of the research on aquatic cycling without focusing on certain details of the included studies. For example, only the main outcomes reported in the abstract of the included studies were reported in this review. Yet, this comprehensive outline of available literature in this scoping review could serve as a starting point for systematic reviews or clinical studies on the effects of aquatic cycling on the cardiovascular responses.

## Conclusion

This is the first scoping review to summarise the literature on head-out aquatic cycling. There are numerous variables related to aquatic cycling e.g., the type of aquatic bike or environmental factors e.g., water temperature or immersion level. As a result, the objectives of the identified studies in this review are heterogeneous. Most of the included studies compared aquatic cycling with land-based cycling or examined how to quantify and modify exercise intensity. Very few studies evaluated the effect of aquatic cycling interventions. Cardiovascular parameters were investigated by many of the studies and the results suggest that the cardiac demand of aquatic cycling seems similar to land-based cycling. Therefore, further research should synthesize the effects of aquatic cycling on cardiovascular parameters in a systematic review. Future studies should evaluate the effects of aquatic cycling interventions in a clinical and rehabilitative context.

## Supporting information

S1 File Post-hoc analysis of search terms. (DOCX)

S2 File Development of the inclusion and exclusion criteria. (DOCX)

S3 File Excluded articles. (DOCX)
