# Supplementary material for: Aquatic cycling—What do we know? A scoping review on head-out aquatic cycling
Source: PLoS One. 2017 May 16;12(5):e0177704. doi: 10.1371/journal.pone.0177704 (PMC5433763; doi:10.1371/journal.pone.0177704)
Supplement: S1 File — (DOCX) [file pone.0177704.s001.docx]

**Post-hoc analysis of search terms**

Across all included articles it was explored how the exercise activity and the water exercise device (ergometer) were described. The majority (84%) of the studies described the type of exercise in a specific manner rather than using general expressions such as “water exercise” or “immersed leg exercise”. Also the word “exercise” (37%) or “cycling” (32%) was combined with a term for the exercise device and the exercise environment. Examples of these descriptions are “exercise on a bicycle ergometer (during immersion) in water”, “cycling in water immersion” or “water cycling exercise”. Nine expressions (15%) were found that extended description by specifying the exercising limb (e.g. performing leg cycle exercise in water) or the body position on the exercise device resulting in descriptions such as “upright cycling exercises in water”.

Likewise to the exercise activity descriptions, most authors (82%) clearly indicate that the device is used in water. In addition, the term “ergometer” is frequently used (68%) and combined with the word “(bi)cycle”. This results in descriptions such as “(bi)cycle ergometer immersed/used in water” , “(under)water (bi)cycle ergometer” or “immersible/submersible ergometer”. In recent publications of Garzon et al. the short description “immersible ergocycle” was introduced. Other descriptions like “whole body ergometer” or “arm / leg ergometer for use in water” focus on the exercising limb(s) or emphasize that the ergometer can be used on land and in water by naming the device an “air-water ergometer”. From 2007 onwards the terms “aqua(tic) bike”, “water (stationary) bike” or “aqua cycle” are occasionally used and account for 13% of all identified descriptions.
