# Supplementary material for: Aquatic cycling—What do we know? A scoping review on head-out aquatic cycling
Source: PLoS One. 2017 May 16;12(5):e0177704. doi: 10.1371/journal.pone.0177704 (PMC5433763; doi:10.1371/journal.pone.0177704)
Supplement: S2 File — (DOCX) [file pone.0177704.s002.docx]

**Supplementary File 2:** Development of the inclusion and exclusion criteria

The inclusion and exclusion criteria were developed in two stages. Prior to screening any articles, the authors agreed to include all formats of full-text research reports that focused on the effects of head-out immersed cycling on the human body (Table 2, stage one). Studies that investigated the effect of full-body immersed underwater cycling, possibly in combination with a self-contained underwater breathing apparatus (SCUBA), were excluded. Two researchers (BW, SR) screened all titles and abstracts to select articles for full-text review. The independent screening and selection of articles was done with the online programme ‘Covidence’ (Covidence systematic review software, Veritas Health Innovation, Melbourne, Australia, available at [www.covidence.org](http://www.covidence.org/)). Next, to become familiar with the literature and to check whether the in- and exclusion criteria needed more specification a pilot full-text review of articles identified from scientific databases (n=68) was carried out. To save time and to stimulate discussion the pilot full-text screening was split between different reviewers. The six reviewers have different backgrounds in physical therapy (AFL, RB, BW, SR), epidemiology (RB, IM), exercise physiology (JB) and aquatic therapy (BW, SR). Thirteen to 14 full-text versions were screened per reviewer and one reviewer (SR) screened all articles. Disagreement between the reviewers concerned whether or not to include studies that do not focus on aqua cycling as an exercise activity, but to study physiological responses as body temperature to immersion. Consequently, the reviewers agreed that these studies might contain useful information as long as the experiments were carried out under usual exercise conditions. Furthermore, the reviewers decided not to exclude a certain type of ergometer. However, the limbs had to be immersed during exercise and the subjects should be seated in upright or semi recumbent position during the exercise. All inclusion and exclusion criteria are summarized in Table 2. Finally, two reviewers (SR, AFL) used the extended list of inclusion on all identified full-text articles.
