# Supplementary material for: Aquatic cycling—What do we know? A scoping review on head-out aquatic cycling
Source: PLoS One. 2017 May 16;12(5):e0177704. doi: 10.1371/journal.pone.0177704 (PMC5433763; doi:10.1371/journal.pone.0177704)
Supplement: S3 File — (DOCX) [file pone.0177704.s003.docx]

**S3. Documentation of exclusion of publications during full-text screening.**

| **Exclusion criteria** | **Study title** | **First author** | **Date** |
| --- | --- | --- | --- |
| Unusual aquatic exercise conditions:   - above head immersion, - long duration immersion - very cold (<10°C) or hot (>41°C) water - manipulation of participants’ homeostasis | Insulation and body temperature of prepubescent children wearing a thermal swimsuit during moderate-intensity water exercise | Wakabayashi H | 2007 |
|  | The effects of hyperthermia and hypoxia on ventilation during low-intensity steady-state exercise | Chu AL | 2007 |
|  | Short-term vasomotor adjustments to post immersion dehydration are hindered by natriuretic peptides. | Mourot L | 2004 |
|  | Cytokine induction during exertional hyperthermia is abolished by core temperature clamping: neuroendocrine regulatory mechanisms | Rhind SG | 2004 |
|  | Metabolic habituation following repeated resting cold-water immersion is not apparent during low-intensity cold-water exercise | Stocks JM | 2001 |
|  | Telemetry pill measurement of core temperature in humans during active heating and cooling | O’Brien C | 1998 |
|  | Thermal and metabolic responses to cold-water immersion at knee, hip, and shoulder levels | Lee DT | 1997 |
|  | Effect of physical exercise on renal response to head-out water immersion | Rim H | 1997 |
|  | Endurance exercise with and without a thermal clamp: effects on leukocytes and leukocyte subsets | Cross MC | 1996 |
|  | Alcohol lowers the vasoconstriction threshold in humans without affecting core cooling rate during mild cold exposure | Johnston CE | 1996 |
|  | Hypercapnia lowers the shivering threshold and increases core cooling rate in humans | Johnston CE | 1996 |
|  | Eucapnic hypoxia lowers human cold thermoregulatory response thresholds and accelerates core cooling | Johnston CE | 1996 |
|  | Effect of hypoglycemia on thermoregulatory responses | Passias TC | 1996 |
|  | Responses to underwater exercise in scuba divers differing in trait anxiety | Raglin JS | 1996 |
|  | Metabolic changes during cold water immersion | Hesselberg O | 1995 |
|  | Human temperature regulation during narcosis induced by inhalation of 30% nitrous oxide | Mekjavic IB | 1992 |
|  | Plasma volume shifts with immersion at rest and two exercise intensities | Ertl AC | 1991 |
|  | Changes in insulation of body tissue and wet suits during underwater exercise at various atmospheric pressures | Iwamoto J | 1990 |
|  | The relationship between ventilation and oxygen consumption in man is the same during both moderate exercise and shivering. | Newstead CG | 1987 |
|  | Changes in thermal insulation during underwater exercise in Korean female wet-suit divers. | Yeon DS | 1987 |
|  | Decrease in body insulation with exercise in cool water | Park YS | 1984 |
|  | Superficial shell insulation in resting and exercising men in cold water. | Veicsteinas A | 1982 |
|  | Influence of water temperature on thermal, circulatory and respiratory responses to muscular work | Pirnay F | 1977 |
|  | Succinate accumulation in man during exercise | Hochachka PW | 1976 |
| Insufficient information about the aquatic cycling intervention | Aquatic circuit training including aqua-cycling in patients with knee osteoarthritis: A feasibility study. | Rewald S | 2015 |
|  | Entwicklung und Überprüfung eines Aqua-Cycling-Programms für Rheumapatienten [German, Masterthesis] | Moser S | 2009 |
|  | Eine retrospektive Studie mit und ohne Unterwasserfahrrad an implantierten Kniegelenksprothesen [German, Dissertation] | Ulatkowski M | 2009 |
|  | Cardiovascular responses to head-out water immersion in Korean women breath-hold divers. | Yun SH | 2004 |
|  | Cardiovascular regulation during water immersion. | Park KS | 1999 |
|  | Prospektiv-randomisierte Vergleichsstudie zur Rehabilitation vorderer Kreunzbandplastiken zwischen koventioneller Therapie und Unterwasserfahrrad [German, Dissertation] | Von Kathen M | 1999 |
|  | Core temperature "null zone" | Mekjavic IB | 1991 |
|  | Determination of stroke volume and cardiac output during exercise: comparison of two-dimensional and Doppler echocardiography, Fick oximetry, and thermodilution | Christie J | 1987 |
| Exercising limb not immersed | Including arm exercise during a cold water immersion recovery better assists restoration of sprint cycling performance | Crampton D | 2014 |
|  | Power output validation during incremental exercise on an immersible ergocycle | Leone M | 2014 |
|  | The heart rate increase at the onset of high-work intensity exercise is accelerated by central blood volume loading | Miyamoto T | 2006 |
|  | Blood lipids of cardiac patients after acute exercise on land and in water | Bermingham MA | 2004 |
|  | Water immersion delays the oxygen uptake response to sitting arm-cranking in humans | Hayashi N | 1999 |
| Article provides a summary of information provided elsewhere | Hemodynamic requirements and thoracic fluid balance during and after 30 minutes immersed exercise: Caution in immersion rehabilitation programmes | Bréchat PH | 2012 |
|  | Vergleichende Untersuchungen von Leistungs- und Stoffwechselparametern im ergometrischen Test an Land und im Wasser [German, Dissertation] | Karnahl B | 2010 |
|  | Cardio-respiratory physical training in water and on land | Avellini BA | 1983 |
| Reviews | Physiological assessment of head-out aquatic exercises in healthy subjects: a qualitative review | Barbosa TM | 2009 |
|  | Heart rate and perceived exertion at aquatic environment: differences in relation to land environment and applications for exercise prescription – a review | Graef F | 2006 |
|  | Unterschiede in der maximalen Sauerstoffaufnahme bei körperlichen Belastungen im Wasser oder an Land | Fenzl M | 2005 |
|  | Wassertherapie bei Herzinsuffizienz | Meyer K | 2005 |
|  | Exercise in heart failure: should aqua therapy and swimming be allowed? | Meyer K | 2004 |
|  | Exercise in water during pregnancy. | Katz | 2003 |
|  | Special ergometric techniques and weight reduction. | Sheldahl | 1986 |
|  | Influence of Body Mass, Morphology and Gender on Thermal Responses during Immersion in Cold Water | Pandolf KB | 1986 |
| Wrong intervention | A comparison of post-match recovery strategies in youth soccer players | Kinugasa T | 2009 |
|  | Energy balance of locomotion with pedal-driven watercraft | Zamparo P | 2008 |
|  | High intensity physical group training in water--an effective training modality for patients with COPD | Wadell K | 2004 |
|  | Energy requirements during exercise in the water | Costill DL | 1971 |
